# Supplementary material for: Causal interplay between lactose intolerance and gut microbiota: a combined bidirectional Mendelian randomization and in vivo validation study
Source: Front Nutr. 2026 Jun 1;13:1803337. doi: 10.3389/fnut.2026.1803337 (PMC13265576; doi:10.3389/fnut.2026.1803337)
Supplement: Supplementary file 1 [file Data_Sheet_1.zip › supplementary materials/Forward/leave one out cross validation/ebi-a-GCST90027595.finngen_R12_E4_LACTONAS.pdf]

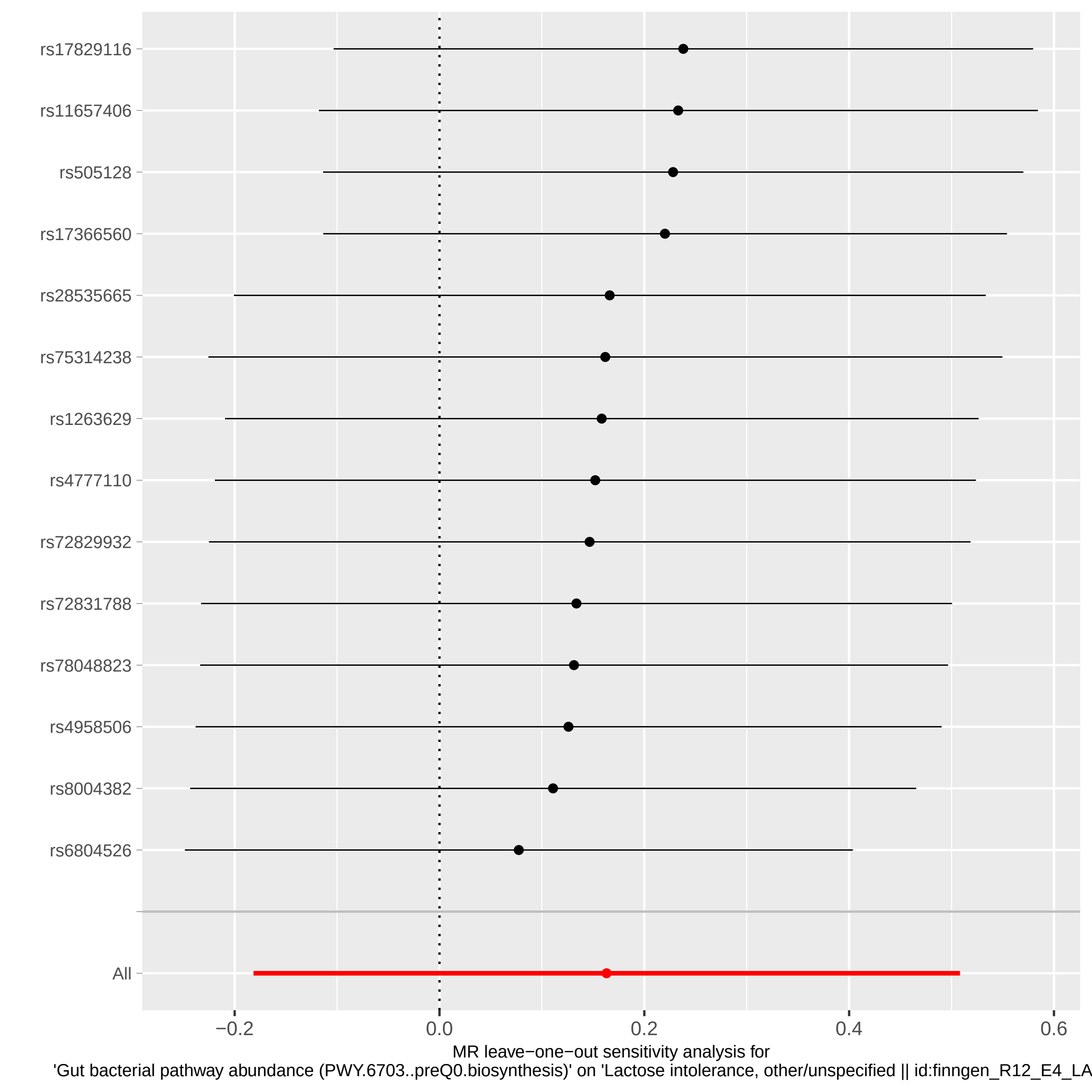
rs17829116
rs11657406
rs505128
rs17366560
rs28535665
rs75314238
rs1263629
rs4777110
rs72829932
rs72831788
rs78048823
rs4958506
rs8004382
rs6804526
All
−0.2
0.0
0.2
0.4
0.6
MR leave−one−out sensitivity analysis for
'Gut bacterial pathway abundance (PWY.6703..preQ0.biosynthesis)' on 'Lactose intolerance, other/unspecified || id:finngen_R12_E4_LA
